# *CTNNB1*-related neurodevelopmental disorder mimics cerebral palsy: case report

**DOI:** 10.3389/fped.2023.1201080

**Published:** 2023-06-16

**Authors:** Jaewoong Lee, Jaeeun Yoo, Seungok Lee, Dae-Hyun Jang

**Affiliations:** ^1^ Department of Laboratory Medicine, Incheon St. Mary’s Hospital, College of Medicine, The Catholic University of Korea, Seoul, Republic of Korea; ^2^ Department of Rehabilitation Medicine, Incheon St. Mary’s Hospital, College of Medicine, The Catholic University of Korea, Seoul, Republic of Korea

**Keywords:** *CTNNB1*, neurodevelopmental disorder, infant, cerebral palsy, mimic

## Abstract

While somatic gain-of-function mutations in the *CTNNB1* gene cause diverse malignancies, germline loss-of-function mutations cause neurodevelopmental disorders or familial exudative vitreoretinopathy. In particular, *CTNNB1*-related neurodevelopmental disorders have various phenotypes, and a genotype-phenotype relationship has not been established. We report two patients with *CTNNB1*-related neurodevelopmental disorder whose clinical features were similar to those of cerebral palsy, hindering diagnosis.

## Introduction

Cerebral palsy (CP) is a group of non-progressive disorders of movement and posture that cause activity limitations due to disturbances that occur in the developing fetal or infant brain (1). Motor symptoms of CP include spasticity, weakness, and involuntary movements. CP mimics a number of neurogenetic disorders, which may present with motor symptoms in early childhood, resulting in misdiagnosis (2). Many inborn errors of metabolism (3), hereditary spastic paraplegias, and enzyme deficiencies (2) may present as CP mimics.

The CTNNB1 gene is located on chromosome 3p22.1 and encodes beta-catenin, which plays a crucial role in cell adhesion in the Wnt signaling pathway. Somatic gain-of-function pathogenic variants in *CTNNB1* cause various malignancies. Recently, germline loss-of-function pathogenic variants in *CTNNB1* have been reported to cause neurodevelopmental disorders with spastic diplegia and visual defects (NEDSDV, MIM# 615075) (4), and familial exudative vitreoretinopathy (MIM# 617572) (5). The phenotypes reported in NEDSDV are global developmental delay, impaired intellectual development, axial hypotonia, spasticity, and dysmorphic craniofacial features with microcephaly. Moreover, the majority of such patients express visual abnormalities, including strabismus, optic nerve atrophy, and retinal abnormalities (6). Here we report two cases of NEDSDV that presented as CP mimics without definite ophthalmologic symptoms. This study was performed in accordance with the ethical standards of the Declaration of Helsinki and was approved by the Institutional Review Board of Incheon St. Mary's Hospital (OC23ZISI0001).

## Case 1

A 17-month-old girl was referred to the Medical Genetics and Rare Disease Center for developmental delay, spasticity, dystonia, and failure to thrive. The patient was delivered by cesarean section due to distress during labor at 40 weeks of gestation. Her birth weight was 2.38 kg, and her family history was nonspecific. The patient was the fourth child, and all the older siblings developed normally. The patient's growth parameters were height, 74 cm (3rd centile); weight, 7.1 kg (3rd centile); and head circumference, 39 cm (3rd centile). She had mild dysmorphic features of thin upper lip, ear abnormality, upslanting palpabral fissures, and flat philtrum. On the Bayley Scales of Infant and Toddler Development (3rd edition), her development corresponded to an equivalent age of 4 months in cognition, 9 months in receptive language, 9 months in expressive language, 3 months in fine motor skills, and 2 months in gross motor skills. Overall, she displayed profound global developmental delay. Brain magnetic resonance imaging was normal. Chromosome analysis, chromosome microarray (CMA), *MECP2* gene sequencing, Prader-Willi syndrome, and Angelman syndrome tests were carried out but revealed normal results. The patient was able to sit up on his own after the age of 2 years. The patient was followed under the diagnosis of dyskinetic CP. On follow-up at the age of 41 months, her development corresponded to an equivalent age of 8 months in cognition, 5 months in receptive language, 6 months in expressive language, 8 months in fine motor skills, and 7 months in gross motor skills. She showed severe developmental delay, dyskinetic movement, and spasticity; however, no definite abnormalities were noted on brain MRI. Because of the discrepancy of clinical manifestations and radiologic findings, a next-generation sequencing (NGS) test for hereditary developmental delay was performed.

### Molecular analyses

A peripheral blood sample was used for genomic DNA extraction, and the TruSight One Extended Sequencing Panel Kit was used for sequencing on a NextSeq 550Dx instrument (Illumina, San Diego, CA). A heterozygous nonsense mutation, NM_001904.3:c.1543C > T, p.Arg515*, was found in *CTNNB1*, which was previously reported as a pathogenic variant. At clinical reassessment, the patient presented global developmental delay, dysmorphic craniofacial features, truncal hypotonia, spasticity, dystonia, failure to thrive, and microcephaly. The patient had no definite ophthalmological symptoms, such as strabismus or vitreous retinopathy, but other phenotypes corresponded to *CTNNB1*-related neurodevelopmental disorders. Thus, NEDSDV was diagnosed.

## Case 2

A 12-month-old girl was referred to the Medical Genetics and Rare Disease Center due to gross motor developmental delay and spasticity. The patient had been born at 38 weeks of gestation without any complications. She was the first child born to non-consanguineous parents. Her birth weight was 2.55 kg, and there were no perinatal problems or specific family history of interest. The patient's growth parameters were height, 70 cm (10th centile); weight, 7.8 kg (3rd centile); and head circumference, 41 cm (below the 1st centile). She had no definite dysmorphic features and could roll over but could not sit alone, even with support. On neurological examination, the deep tendon reflexes of both the knee and ankle were exaggerated. Additionally, primitive reflexes were exaggerated. Spasticity was prominent in both lower limbs and was rated as Modified Ashworth Scale Grade I+. Dyskinetic movement was not definite. On Bayley Scales of Infant and Toddler Development (3rd edition), her development corresponded to an equivalent age of 4 months in cognition, 4 months in receptive language, 9 months in expressive language, 4 months in fine motor skills, and 4 months in gross motor skills. Brain magnetic resonance imaging was normal. Due to the discrepancy of clinical manifestations and radiologic findings, Chromosome analysis, CMA, and NGS tests for hereditary developmental delay were simultaneously performed.

### Molecular analyses

**Figure 1:**
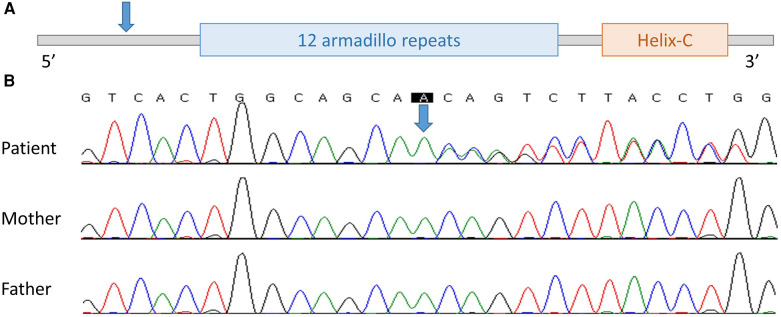
The location of novel variant on *CTNNB1* found in case 2. (**A**) Schematic location of the variant on *CTNNB1*. (**B**) Electropherogram of the patient, patient's mother, and patient's father (from the top). Blue arrow, the location of the variant.

No abnormalities were observed in chromosome analysis or CMA. In NGS, a heterozygous novel duplication variant, NM_001904.3:c.81dup, p.Gln28Thrfs*22, was found in *CTNNB1* and was confirmed by Sanger sequencing. Sequencing was performed with a tailored primer pair, and the Primer3Plus webpage (http://www.bioinformatics.nl/cgi-bin/primer3plus/primer3plus.cgi) was used for primer design. The patient's symptoms including global developmental delay, spasticity, and microcephaly were consistent with NEDSDV. There was no visual abnormality, such as strabismus or optic nerve atrophy, so this patient was thought to have CP until the NGS results were received. In Sanger sequencing of the patient's asymptomatic parents, the variant was not found and was confirmed to be *de novo* (Figure 1). The variant observed in the second patient has not been previously reported but was classified as “pathogenic” according to the 2015 ACMG/AMP guidelines (PVS1 + PS2_supporting + PM2_supporting) (7). Thus, we registered this novel variant as pathogenic in ClinVar (accession: VCV001879838.1).

## Discussion

Diseases known as CP mimics are very diverse and include a number of metabolic and genetic disorders (3, 8). In previous studies, various disease entities and characteristics were listed to overcome these diagnostic difficulties. However, diseases corresponding to CP mimics are being newly discovered, and extremely rare diseases are not included in previous studies. NEDSDV is also not included in the list of diseases to be differentiated in previous studies, and findings suggesting a CP mimic disease are non-specific, hindering differential diagnosis (8). Regression of milestones, isolated motor dysfunctions, and non-specific brain imaging results are examples of such findings.

NEDSDV was first reported in 2012 (9) and has a incidence of 1 in 50,000 worldwide. In previous reports, the phenotypes of such patients included developmental delay and intellectual disability but included many diverse symptoms (5, 10, 11). In particular, in a previous Korean report, genetic diagnosis was confirmed in children aged 4 years and older, and the delays in language and motor development, presence of strabismus, unilateral persistent hyperplastic primary vitreous (PHPV), and microphthalmia varied by patient (6). The patients in the present study had no visual abnormality in infant vision screening; however, the young age of the patients indicates the need for continuous observation for phenotypic changes. Facial dysmorphism was not very specific in infancy in our cases, which could have contributed to misdiagnosis as CP. A comparison of phenotypes in our cases and those in the literature review is presented in Table 1 (4, 5, 9–34). Known phenotypes with high frequency were motor abnormalities (97%), facial dysmorphisms (91%), ophthalmologic abnormalities (91%), speech difficulties (87%), and microcephaly (71%). Although these phenotypes were frequently reported, individual subtypes of phenotypes and degrees of the phenotypes were diverse among studies. Despite the high frequency of ophthalmologic abnormalities, patients in this study did not show any ophthalmologic abnormality. The facial dysmorphisms shown in case 1 were subtle and were only identified at reassessment after the genetic test results were reported.

**Table 1:** Phenotype comparison of our cases and literature review.

	Literature review^a^	Case 1	Case 2
Facial dysmorphisms^b^	91% (58/64)	+	−
Ophthalmologic abnormalities^c^	91% (96/105)	−	−
Motor abnormalities (CP mimics)^d^	97% (105/108)	+	+
Speech difficulties	87% (66/76)	+	+
Microcephaly^e^	71% (59/83)	+	+

^a^ Literature review includes case reports from 2012 to 2022 (4, 5, 9–34).

^b^ Facial dysmorphisms include small alae nasi, long and/or flat philtrum, thin upper lip vermillion, and broad nasal tip.

^c^ Ophthalmologic abnormalities include strabismus, familial exudative vitreoretinopathy, hyperopia, myopia, astigmatism, esotropia.

^d^ Motor abnormailities (CP mimics) include hypotonia and spasticity.

^e^ Microcephaly is defined as head circumference of 3rd centile or below.

The number of pathogenic/likely-pathogenic variants reported to be associated with NEDSDV in ClinVar is rapidly increasing every year, and they currently number 70 (6, 10, 35).

Pathogenic/likely-pathogenic variants previously reported in ClinVar are distributed across all exons after exon 3 of *CTNNB1*, and there is no specific hot-spot (10). In addition, since a genotype-phenotype relationship has not been established, it is difficult to infer the phenotype through genetic mutation (36). As shown in this case, NEDSDV in infants may show nonspecific developmental delay and microcephaly without characteristic ophthalmic manifestations (5, 10, 11, 35), leading to confusion with CP, although eye abnormalities are found in 91% of NEDSDV cases (37). Thus, screening tests such as targeted panel sequencing or whole exome sequencing should be considered for early differential diagnosis.

## Conclusion

We described two NEDSDV cases with genetically confirmed pathogenic variants in *CTNNB1*. These cases presented phenotypes as CP mimics and eventually were diagnosed as NEDSDV in infancy without definite ophthalmologic manifestations. This report highlights the importance of consideration of *CTNNB1*-related neurodevelopmental disorder in differential diagnosis of CP mimics in infants.

## Data Availability

The original contributions presented in the study are included in the article, further inquiries can be directed to the corresponding authors.
